# Ca^2+^/Calmodulin Complex Triggers CAMTA Transcriptional Machinery Under Stress in Plants: Signaling Cascade and Molecular Regulation

**DOI:** 10.3389/fpls.2020.598327

**Published:** 2020-12-03

**Authors:** Zahra Iqbal, Mohammed Shariq Iqbal, Surendra Pratap Singh, Teerapong Buaboocha

**Affiliations:** ^1^Molecular Crop Research Unit, Department of Biochemistry, Chulalongkorn University, Bangkok, Thailand; ^2^Amity Institute of Biotechnology, Amity University, Uttar Pradesh, Lucknow Campus, Lucknow, India; ^3^Plant Molecular Biology Laboratory, Department of Botany, Dayanand Anglo-Vedic (PG) College, Chhatrapati Shahu Ji Maharaj University, Kanpur, India; ^4^Omics Sciences and Bioinformatics Center, Faculty of Science, Chulalongkorn University, Bangkok, Thailand

**Keywords:** calcium, calmodulin, development, transcription factor, CAMTA, stress

## Abstract

Calcium (Ca^2+^) ion is a critical ubiquitous intracellular second messenger, acting as a lead currency for several distinct signal transduction pathways. Transient perturbations in free cytosolic Ca^2+^ ([Ca^2+^]_cyt_) concentrations are indispensable for the translation of signals into adaptive biological responses. The transient increase in [Ca^2+^]_cyt_ levels is sensed by an array of Ca^2+^ sensor relay proteins such as calmodulin (CaM), eventually leading to conformational changes and activation of CaM. CaM, in a Ca^2+^-dependent manner, regulates several transcription factors (TFs) that are implicated in various molecular, physiological, and biochemical functions in cells. CAMTA (calmodulin-binding transcription activator) is one such member of the Ca^2+^-loaded CaM-dependent family of TFs. The present review focuses on Ca^2+^ as a second messenger, its interaction with CaM, and Ca^2+^/CaM-mediated CAMTA transcriptional regulation in plants. The review recapitulates the molecular and physiological functions of CAMTA in model plants and various crops, confirming its probable involvement in stress signaling pathways and overall plant development. Studying Ca^2+^/CaM-mediated CAMTA TF will help in answering key questions concerning signaling cascades and molecular regulation under stress conditions and plant growth, thus improving our knowledge for crop improvement.

## Introduction

Plants have evolved several strategies, majorly through molecular mechanisms, to optimize growth and ameliorate tolerance toward environmental constraints (Tripathi et al., 2020). One of the basic mechanisms is an increase in the concentration of [Ca^2+^]_cyt_ in response to various external stimulus (Hepler, 2005). Ca^2+^ signatures are considered as core regulators of many adaptive and developmental processes. They are characterized by stimulus-driven signals resulting from the cumulative action of carriers, pumps, and channels. These transporters drive Ca^2+^ signals spatially and temporally to act inside a cell. Spatial and temporal Ca^2+^ signals are transmitted and decoded by a bunch of Ca^2+^-binding proteins such as calmodulin (CaM) and calcineurin. These proteins further spread relayed information to generate specific downstream response by regulating transcription factors (TFs). TFs are DNA-binding regulatory proteins involved in distinctive expression of genes that regulate developmental processes and environmental stress responses (Hong, 2016; Mitsis et al., 2020). CaM, upon interaction with Ca^2+^, undergoes conformational changes to modulate several TFs (Reddy, 2001). TFs contribute to various facets of cellular processes and act as a toolkit to signals perceived from within and outside an organism (Mitsis et al., 2020). Intriguingly, TFs constitute a major portion of eukaryotic genome, accounting for approximately 5% of the total genome (approximately 2,000 genes) in humans (Tupler et al., 2001). In *Arabidopsis thaliana*, 11.8% of their genome encodes for TFs (approximately 3,000 genes) (Arabidopsis Genome Initiative, 2000). TFs such as DREB (Agarwal et al., 2006; Chen et al., 2008; Lata and Prasad, 2011; Jan et al., 2017), NAC (Tran et al., 2004; Nakashima et al., 2007; Puranik et al., 2012; Marques et al., 2017), bHLH (Sun et al., 2018; Wang et al., 2020), WRKY (Park et al., 2005; Phukan et al., 2016), and MYB (Levy et al., 2005; Roy, 2016; Zhang et al., 2018) had been previously reported to play crucial roles in stress biology and plant growth. In a similar vein, CAMTA (calmodulin-binding transcription activator) in a Ca^2+^/CaM-driven modus had been involved in carrying out important functions by modulating plant stress responses and overall development (Bouche et al., 2002, 2005; Galon et al., 2010a; Liu et al., 2015; Shkolnik et al., 2019). The present review recapitulates the progress made in comprehending the involvement of Ca^2+^/CaM-mediated CAMTA regulation in stress adaptation and plant development.

## Calcium: A Second Messenger and Its Interaction With Calmodulin

A second messenger is a molecule that acts to transmit signals from a receptor to a target (Bush, 1995). Ca^2+^ is one of the best characterized second messengers. It is hypothesized that Ca^2+^, as a messenger ion, emerged early during cell evolution (Edel et al., 2017). The role of Ca^2+^ as a primary nutrient in sustaining the structural integrity of cell walls and modulating diverse physiological processes such as biotic and abiotic stresses had been elucidated well by extensive biochemical and genetic studies (Poovaiah et al., 1993; Zielinski, 1998; Reddy, 2001; Snedden and Fromm, 2001; Sanders et al., 2002; Harper et al., 2004; Reddy et al., 2004; Bouche et al., 2005; Hepler, 2005; Aghdam et al., 2012; Thor, 2019; Tian et al., 2020). Ca^2+^ spikes are usually the outcome of two opposing reactions occurring in cells: Ca^2+^ influx (entry) via dedicated channels or Ca^2+^ efflux (exit) via specific pumps (Xiong et al., 2006; Tuteja and Mahajan, 2007). The regulation of nanomolar concentration of Ca^2+^ is achieved by two mechanisms. First, Ca^2+^-ATPases pump [Ca^2+^]_cyt_ to the exterior or into organelles such as vacuoles and endoplasmic reticulum. Second, the opening of Ca^2+^-permeable ion channels results in the Ca^2+^ influx to the cytosol (Medvedev, 2005; Tuteja and Mahajan, 2007; Swarbreck et al., 2013; Demidchik and Shabala, 2018). Transmembrane Ca^2+^ gradients maintain sufficient energy to drive Ca^2+^ flux passively into the cytosol from the apoplast or the organelles (Medvedev, 2005; Swarbreck et al., 2013; Demidchik and Shabala, 2018).

Much evidence indicates that Ca^2+^-mediated signaling is implicated in the relay of stress signals such as light (Kim et al., 2003; Liang et al., 2009; Hochmal et al., 2015; Hou et al., 2019), temperature (Teige, 2019), salt (Rahman A. et al., 2016; Manishankar et al., 2018; Seifikalhor et al., 2019), cold (Shi et al., 2014; Yuan et al., 2018b), and gravity (Kordyum, 2003; Toyota et al., 2008; Salmi et al., 2011); oxidative signals such as pathogen attack (Kiep et al., 2015; Aldon et al., 2018; Tian et al., 2019) and reactive oxygen species (Mori and Schroeder, 2004; Monshausen et al., 2009; Kurusu et al., 2015); and hormone signals such as ethylene (Li et al., 2018; Zhu et al., 2018), abscisic acid (ABA) (Edel and Kudla, 2016; Chen et al., 2017; Yuenyong et al., 2018), gibberellins (Abbasi et al., 2004; Nakata et al., 2009; Li et al., 2013), and auxins (Vanneste and Friml, 2013; Hazak et al., 2019). In plants, oodles of Ca^2+^-binding proteins function as Ca^2+^ sensors decoding complex Ca^2+^ signatures (Kudla et al., 2018). These binding proteins sense changes in [Ca^2+^]_cyt_ and/or [Ca^2+^]_nuc_, regulating downstream signaling processes and hence drawing physiological response against them (Day et al., 2002; Boonburapong and Buaboocha, 2007). Ca^2+^ sensors are basically proteins with highly conserved one or multiple helix-turn-helix structures-EF-hand (Nakayama et al., 2000; Day et al., 2002). Approximately, 250 EF-hand comprising putative Ca^2+^ sensors are known in *A. thaliana*, which accounts for about 1% of the reported proteome (Day et al., 2002). Ca^2+^ sensors are divided into three categories, namely, (i) CaM and CMLs (calmodulin-like proteins) (Bender and Snedden, 2013), (ii) CBLs (calcineurin-B-like proteins) (Luan, 2009), and (iii) CPKs (Ca^2+^-dependent protein kinases) and CCaMK (calcium and calcium/calmodulin-dependent protein kinase) (Cheng et al., 2002).

**FIGURE 1:**
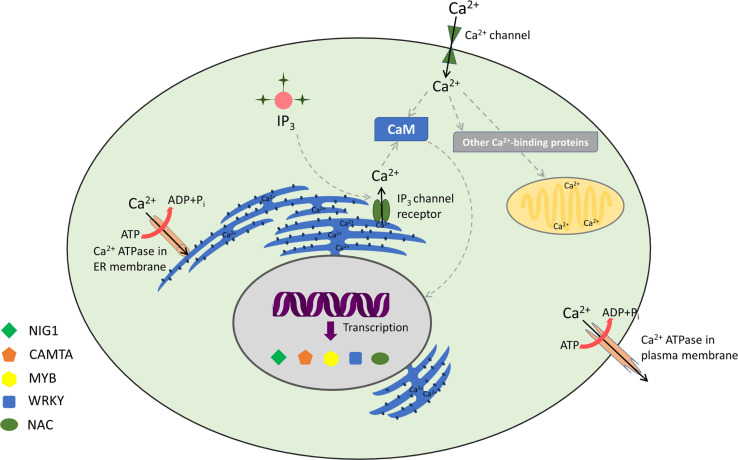
Decoding of calcium signatures: calcium signaling and its sensors with associated transcription factors.

Calmodulins are a class of extensively studied Ca^2+^ sensors and are known to regulate diverse cellular processes in plants, including stress responses and plant development (Zielinski, 1998; Bouche et al., 2002; Bergey et al., 2014; Zeng et al., 2015; Dubrovina et al., 2019). CaM is a 17-kDa dumbbell-shaped cytosolic acidic protein, with a supple joint in the middle (Meador et al., 1992). CaMs contain four EF-hand motifs and are highly conserved among eukaryotes (Zielinski, 1998; Reddy et al., 2002). It has been ascertained that around 300 proteins interact with CaMs in plants (Reddy et al., 2002; Bouche et al., 2005; Popescu et al., 2007). Among the known protein–protein interactions, CaMs are implicated to have maximum interacting partners (Lee et al., 2010). Conformational changes upon Ca^2+^/CaM association lead to contact of hydrophobic surface within each domain (Liu et al., 2017). This ultimately elevates the CaM’s Ca^2+^ sensor bustle, thus resulting in an interaction with its target proteins (Snedden and Fromm, 2001). CaM can act either directly by interacting with key target enzymes or indirectly with the help of specific kinases (Zielinski, 1998; Mt and Mn, 2008; Villalobo et al., 2019). Interestingly, Ca^2+^ ions interact with CaM in a cooperative manner, such that even small changes in the level of [Ca^2+^]_cyt_ lead to huge alteration in the levels of active CaM (Beccia et al., 2015; Fernandes and Oliveira-Brett, 2017). Various CaM proteins display differential expression and possess variable affinity to Ca^2+^, including their downstream target proteins (McCormack et al., 2005; Popescu et al., 2007; Chinpongpanich et al., 2015). Many Ca^2+^- and Ca^2+^/CaM-associated TFs, implicated in stress signaling, are found in plants (Singh and Virdi, 2013; Shen et al., 2020). However, few TFs are specifically transcribed to distinct Ca^2+^ signal durations and amplitude. For example, the binding of Ca^2+^ can directly regulate the activity of certain TFs such as the DREAM (downstream regulatory element antagonist modulator) protein (Carrion et al., 1999). Similarly, *AtNIG1* (*A. thaliana NaCl-inducible gene 1*) is also classified as a Ca^2+^-dependent TF, which is targeted in the nucleus, indicating that it is a nuclear Ca^2+^-binding protein (Kim and Kim, 2006). Taking Ca^2+^/CaM-associated TFs into account, *AtGT2L*, a member of GT-2 subfamily, upon interaction with Ca^2+^/CaM, is responsive to freezing and salinity stresses in plants (Xi et al., 2012). WRKY is also implicated to be a Ca^2+^/CaM-dependent gene (Park et al., 2005; Yan et al., 2018). *AtWRKY7* is well reported to be involved in Ca^2+^/CaM-dependent gene regulation and has a role in pathogen incursion (Park et al., 2005). Experimental results have revealed that CaM binds exclusively to the Ca^2+^-regulated CaM-binding domain (CaMBD) of *AtWRKY7* (Park et al., 2005). In addition, WRKY45, WRKY43, WRKY53, and WRKY50 bind to various isoforms of CaM in a Ca^2+^-driven behavior (Popescu et al., 2007). Myb is another TF that is well characterized as a Ca^2+^/CaM-dependent protein. It acts upstream to a number of defense-responsive, salinity, and drought-receptive genes (Do Heo et al., 1999; Stracke et al., 2001; Li et al., 2019). CaM also supports transcriptional repression interceded by CBNAC/NTL9 (Kim et al., 2007). The TF *ZmNAC84* has been reported to interact physically with *ZmCCaMK* both *in vivo* and *in vitro*. *ZmNAC84*, after ABA induction, has a partially overlapping expression profile with *ZmCCaMK.* Its overexpression renders drought tolerance and resistance to oxidative stress induction owing to drought (Zhu et al., 2016). Moreover, Cam7, also known as ZBF3, is a CaM isoform in *A. thaliana* that works as a transcriptional regulator. The upstream elements to TSS of light-inducible genes are directly targeted by ZBF3 to promote photomorphogenesis (Yadav et al., 2005; Kushwaha et al., 2008). In a similar context, recently, CaM proteins have also been implicated in salt stress. “Khao Dawk Mali 105” rice variety, which overexpressed *OsCam1-1*, shows differential expression of several genes implicated in salt stress signaling, hormonal regulation, lipid/carbohydrate/secondary metabolism, photosynthesis, etc. Owing to *OsCam1-1* overexpression, the rate of photosynthesis declines in transgenic rice, whereas content of sucrose and starch increases under salt stress. This study further revealed that CaM under salt stress condition boosts several metabolic enzymes implicated in energy pathways of plant cells, which either conserve or generate energy under limited photosynthesis (Yuenyong et al., 2018). Thus, Ca^2+^/CaM acts as an intermediate complex between stimulus perception and gene expression (Figure 1).

## Calmodulin-Binding Transcription Activator

Calmodulin-binding transcription activator also known as SR (signal responsive) protein (Yang and Poovaiah, 2000) or EICBP (ethylene-induced CaM-binding proteins) (Reddy et al., 2000) is considered as the largest and best characterized family of CaM-binding TFs (Finkler et al., 2007; Kim et al., 2009). The presence of this novel protein that binds to DNA was for the very first time identified during the process of isolation of partial cDNA clone (CG-1) from parsley (*Petroselinum crispum*) (e Silva, 1994). It was shown that CGCG was the signature motif required for the binding of this protein to DNA. Upon exposure to UV-containing white light, the CG-1 coding mRNA was rapidly and transiently accumulated in parsley cultured cells; however, until then, CG-1 was not designated as a Ca^2+^/CaM-binding protein (e Silva, 1994). Later, Yang and Poovaiah (2000) identified NtER1 as a CaM-binding protein engaged in ethylene-regulated plant death and senescence, indicating its potential application in prolonged storage life of horticultural crops. They also showed that CaM binds with a very high affinity (*Kd*∼12 nM) in a Ca^2+^-dependent manner to NtER1 (Yang and Poovaiah, 2000). Yang and Poovaiah (2002) further reported a homolog of *NtER1* and five related genes in *Arabidopsis* (*AtSRs*). Their results evidently suggested *AtSRs* as a CaM-binding gene family with CGCG as a core motif in their promoters. The study also indicated probable implication of CAMTAs in numerous signal transduction pathways in plants (Yang and Poovaiah, 2002). Since then, CAMTA TF had been reported in various multicellular organisms. With the advancement in computational techniques, proteins similar to CAMTA had been reported in unicellular eukaryotes such as the ciliates, *Paramecium tetraurelia* and *Tetrahymena thermophila* (Finkler et al., 2007). The orthologs of CAMTA had also been reported in *Arabidopsis* (Yang and Poovaiah, 2002; Bouche et al., 2005); humans (Song et al., 2006); flies (Han et al., 2006); and in various crop plants such as tobacco (Yang and Poovaiah, 2000), rice (Choi et al., 2005), tomato (Yang et al., 2012), grapevine (Shangguan et al., 2014), corn (Yue et al., 2015), strawberry (Leng et al., 2015), barrelclover (Yang et al., 2015), soybean (Wang et al., 2015), poplar (Wei et al., 2017), and cotton (Pant et al., 2018). Deciphering the CAMTA gene expression patterns and functional redundancies in model plants and various crops laid a foundation for further investigation of its role, which can be beneficially applied to agricultural sector. CAMTA as a TF has been extensively studied for its involvement in stress and developmental biology (Galon et al., 2008; Doherty et al., 2009; Pandey et al., 2013; Sun et al., 2020) (discussed in *CAMTA Transcriptional Response Under Abiotic Stress Conditions* and *CAMTA Transcriptional Response Under Biotic Stress Conditions*).

The most important characteristic of this TF is its affinity for CaM, indicating its participation in Ca^2+^ sensing. Ca^2+^ signatures are effectively decoded by CAMTAs to generate explicit gene expression patterns (Liu et al., 2015). Liu et al. deciphered how different Ca^2+^ signatures are decoded by CAMTAs for specific gene expression patterns. The group devised a dynamic model based on thermodynamic and kinetic principles for understanding the Ca^2+^–CaM–CAMTA binding and the subsequent gene expression patterns. The modeling analysis unraveled that Ca^2+^ signals in the form of elevated [Ca^2+^]_cyt_ are non-linearly amplified upon binding of Ca^2+^, CaM, and CAMTAs. They further combined experimental data with mathematical modeling to comprehend the information flow from Ca^2+^ signatures to CAMTA−regulated gene expression patterns (Liu et al., 2015). This study provided great insight about the involvement of Ca^2+^/CaM in regulation of CAMTAs. The succeeding section focuses on the transcriptional machinery of CAMTA with respect to its molecular and physiological functions in regulating stress responses and overall plant development.

### CAMTA Domain Organization

**FIGURE 2:**
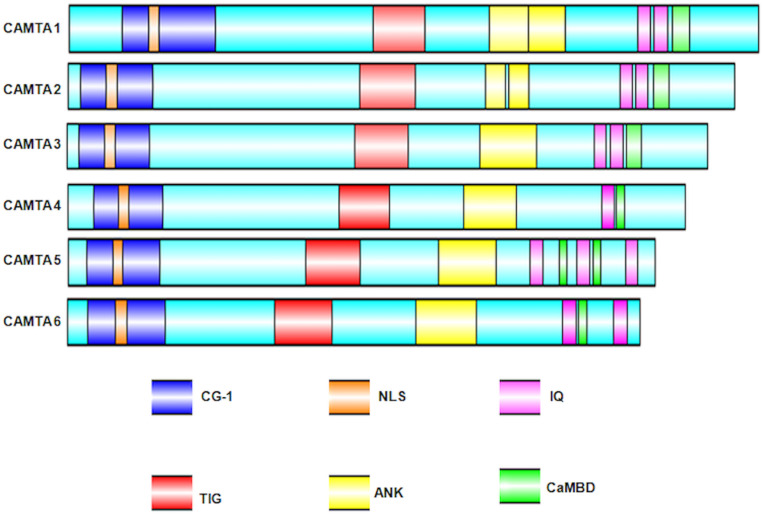
Schematic representation of the *Arabidopsis thaliana* CAMTA domain organization. Domain organization was obtained using NCBI/BLAST/CD-SEARCH (https://www.ncbi.nlm.nih.gov/Structure/cdd/wrpsb.cgi). NLS was specifically searched in Motif scan (http://myhits.isb-sib.ch/cgi-bin/motif_ scan). CaMBD were searched using Calmodulin Target Database (http://calcium.uhnres.utoronto.ca/ctdb/ctdb/). The domain structures were drawn using Illustrator for Biological sequences software (http://ibs.biocuckoo.org/).

Calmodulin-binding transcription activators were reported to consist of multiple functional domains (Finkler et al., 2007). These domains have evolutionary conserved amino acid sequence, organized into a specific conserved order. The predicted functional domains of CAMTA include (a) NLS (nuclear localization signals) for targeting protein into nucleus. These signals have been reported in almost all the CAMTAs, but their localization varies in different organisms; (b) CG-1 domain, which is a unique domain implicated in DNA binding; for any protein to be characterized as CAMTA, the presence of CG-1 domain is obligatory; (c) TIG, which is involved in non-specific DNA interactions in TFs (Aravind and Koonin, 1999) and also implicated in protein dimerization (Muller et al., 1995); (d) ankyrin (ANK) repeats, which are tandem repeats of about 33 amino acids in a variety of eukaryotic and viral proteins and contribute in protein–protein interactions (Sedgwick and Smerdon, 1999; Rubtsov and Lopina, 2000); (e) CaMBD, which is implicated in the association of Ca^2+^-loaded CaM to CAMTAs; (f) IQ motifs, which are implicated in the binding of CaM and CaM-like proteins and are regions of low complexity with a consensus motif IQXXXRGXXX (Bahler and Rhoads, 2002). In some cases, the presence of transcription activation domains (TADs) has also been mapped. The presence of TAD was reported in *AtCAMTA1* (Bouche et al., 2002), *HsCAMTA2* (Song et al., 2006), and *DmCAMTA* (Han et al., 2006). However, owing to lack of sequence homology in this domain, it cannot be inferred that all CAMTAs possess TAD. All these domains are pivotal for the transcription of CAMTA. Nonetheless, certain variations exist; for example, the mapping of Ca^2+^-loaded CaM domain on *AtCAMTA1* reveals a binding site adjacent to IQ motif, indicating multiple CaM binding sites (Bouche et al., 2002). Similarly, in *OsCAMTA*, the Ca^2+^-dependent CaMBD and Ca^2+^-independent CaM dissociation domains were mapped on C-termini (Choi et al., 2005). Recently, in *Gossypium* species, *GrCAMTA5.2*, *GrCAMTA5.3*, and *GhCAMTA3D.1*, *GhCAMTA5D.1* were depicted as TIG lacking CAMTAs in *Gossypium raimondii* and *Gossypium hirsutum*, respectively (Pant et al., 2018). Similar to *Gossypium* study, five TaCAMTAs (*Triticum aestivum* CAMTA), namely, *TaCAMTA1-A/B/D* and *TaCAMTA5-A/D*, also lacked TIG domain (Yang et al., 2020). The domain organization of six *Arabidopsis* CAMTAs, analyzed bioinformatically, is shown in Figure 2.

### CAMTA Transcriptional Response Under Abiotic Stress Conditions

The underpinning mechanisms of CAMTA under abiotic stress conditions had been reasonably elucidated (Pandey et al., 2013; Noman et al., 2019; Shkolnik et al., 2019). Under normal conditions, where plants are not exposed to any stress, there is minimal induction of CAMTA genes. This could be either because the genes in this family of TFs share functional redundancy or the genes in this family are expressed under certain specific environmental conditions. Taking the model plant *A. thaliana* into account, six CAMTAs were reported: *CAMTA1*, *CAMTA2*, *CAMTA3*, *CAMTA4*, *CAMTA5*, and *CAMTA6*. It was shown that all these genes were rapidly and differentially triggered by environmental cues such as high temperatures, salinity, H_2_O_2_, physical wounds; and hormones such as ethylene, ABA, methyl jasmonate (MeJA), and salicylic acid (SA) (Yang and Poovaiah, 2002; Kim et al., 2013, 2017; Pandey et al., 2013; Shkolnik et al., 2019). Considering the first member of *Arabidopsis* CAMTA gene family, *AtCAMTA1*, its role has been reported in auxin signaling (increased expression of AUX/IAA-IAA29), transport, and homeostasis. For transgenic plants possessing *AtCAMTA1* promoter, GUS construct displayed cell-specific expression profiles of auxin. The involvement of CAMTA1 in auxin signaling is established by chemical impairment of polar auxin transport. Gene expression profiling of auxin transport inhibitor 1-N-naphthylphthalamic acid to plants also supports the responsiveness of CAMTA1 in auxin pathways. Genome-wide transcriptome of *camta1* mutant mapped 63 upregulated genes. Further analysis revealed that 17 genes were involved in auxin signaling. Moreover, on analysis of hypocotyl elongation, the *camta1* mutants were hyperresponsive to auxin exposure as compared to wild type (Galon et al., 2010a). It was further reported that *CAMTA1*, *CAMTA2*, and *CAMTA3* are negative regulators of auxin and are associated with genes responsible for red light and high light responses, whereas *CAMTA4*, *CAMTA5*, and *CAMTA6* are positive regulators of auxins and are associated with genes responsible for blue light and darkness responses (Galon et al., 2010b). In a similar vein, phytohormonal regulation of CAMTAs is also extended to brassinosteroid (BR) signaling in which BZR1 (involved in BR signaling cascade) has CAMTA5 as an associated protein (Wang et al., 2013).

Additionally, *camta1* responded severely to cold stress, indicating its role in cold stress management. CAMTA protein provides a much-needed connecting link between Ca^2+^ signatures and cold acclimatization. CBF TF plays a pivotal role in cold acclimation of plants. In *A. thaliana, CBF* genes induce approximately 100 more genes (termed as CBF regulon) upon exposure to low temperatures (Maruyama et al., 2004; Vogel et al., 2005). CAMTA binds to consensus sequence located in the promoter of the *CBF2* gene. The 1,000-bp upstream region of *CBF2* gene comprises seven conserved consensus motifs: CM1 to CM7. The CAMTA signature domain CG-1 matches the CM2 motif of CBF2. CBF2 expression is impaired in *camta3* mutants and impaired to a much higher extent in *camta1* and *camta3* double mutant. These are indications of the intersecting functions of different CAMTA proteins. Therefore, CAMTA controls CBF regulon, which confers freezing tolerance to plants (Doherty et al., 2009). Moreover, CAMTA1, CAMTA2, and CAMTA3 work in recital to induce CBF1, CBF2, and CBF3 and confer freezing tolerance to plants at low temperatures. Furthermore, at warmer temperatures, these three CAMTAs collectively inhibit SA biosynthesis. It had been previously shown that exposure to low temperatures increases SA levels (Scott et al., 2004). SA biosynthesis at low temperatures involves ICS (isochorismate synthase) pathway, encircling *ICS1*, *CBP60g*, and *SARD1* genes. At warm temperatures, CAMTA1, CAMTA2, and CAMTA3 suppress the accretion of *ICS1*, *CBP60g*, and *SARD1*. Such suppression by CAMTA proteins was not observed at low temperatures (Kim et al., 2013). More neoteric inventions have elucidated that the suppression of genes involved in SA biosynthesis by CAMTA at high temperatures (22°C) is overcome when plants are infected by biotrophic or hemibiotrophic pathogens and grown at low temperatures (4°C) for more than a week. Suppression of SA pathway by CAMTA3 under normal condition involves an N-terminal repression module (NRM). NRM works autonomously of CaM binding to CaMBD or the IQ. This finding is different owing to the fact that CAMTA3 repressional assertion involves the binding of CaM to CaMBD. To sum up, the repression activity of CAMTA3 at low temperatures and pathogen infection is governed by related mechanisms (Kim et al., 2017). To further elaborate, three dehydration-responsive element (DRE) binding protein 1/C-repeat binding factors (DREB1/CBFs) are master regulators of cold-responsive gene expression. DREB1 stimulates cold-responsive transcriptional cascade, eventually inducing several genes implicated in cold stress response. Rapid and gradual temperature decreases are sensed as cold stress by plants, which results in the activation of *DREB1* genes. CAMTA3 and CAMTA5 are responsive to rapid decline in temperatures by inducing the expression of *DREB1s*. However, CAMTA3 and CAMTA5 do not respond to gradual declines in temperatures. In contrast to *circadian clock associated 1* and *late elongated hypocotyl* (acts as transcriptional activators to regulate *DREB1* expression only during the day), *AtCAMTA3* and *AtCAMTA5* function both during the day and night (Kidokoro et al., 2017).

Drought and salinity are considered as few of the worst conditions in agriculture and horticulture. The role of CAMTA1 has been extended to drought response (Pandey et al., 2013). The mutants had impaired photosystem II efficiency and water use efficiency, hence making plants susceptible to drought. The mutants had relatively low water content and retarded growth. Microarray analysis revealed drought revival, osmotic balance, apoptosis, DNA methylation, and photosynthesis as investigative pathways in drought-treated *camta1* mutants. It has also been shown that CAMTA1 regulates a broad spectrum of stress-inducible genes such as *RD26*, *ERD7*, *RAB18*, *LTP*s, *COR78*, *CBF1*, and *HSP*s. To ascertain the claims, CAMTA recognition *cis-*element was found to be enriched in the 1,000-bp upstream regions of the listed genes (Pandey et al., 2013). Very recently, CAMTA6 has been shown to be associated with sodium (Na^+^) homeostasis during early seed germination. The *camta6* mutants accumulate less NaCl and display tolerance to salinity and ABA. *AtHKT1* (high affinity K^+^ transporter 1 encoding a Na^+^/K^+^ transporter) expression was limited to radicles and did not increase upon subjecting *camta6* to salt stress or ABA treatment. In addition, even the transcriptome of CAMTA6 under control and salt stress condition revealed 1,020 upregulated and 1,467 downregulated salt-responsive genes in the wild type (Shkolnik et al., 2019). With regard to context, although CAMTA2 in plants has been less explored, studies highlight the regulation of *AtALMT1* (aluminum-activated malate transporter 1) by CAMTA2 in association with WRKY46 (Tokizawa et al., 2015; Wu et al., 2019). Next, considering the involvement of CAMTAs in plant development, the role of CAMTA1 and CAMTA5 is also extended in pollen development. The AVP1 promoter activity has been reported to be activated by *AtCAMTA1* in cultured cells. *AtCAMTA1* triggers the GUS reporter expression driven by P281 promoters of *AVP1* but does not stimulate the GUS reporter expression in mutants with disrupted despaired CGCG-box, indicating that *AtCAMTA1* attaches to CGCG-box and elevates *AVP1* expression. *AtCAMTA1* in collaboration with *AtCAMTA5*, cooperatively elevates their gene function and enhance *AVP1* expression during pollen development (Mitsuda et al., 2003). Apart from *A. thaliana*, CAMTAs from other plant species have also been implicated very well in abiotic stress responses. Various *in silico* and quantitative reverse transcription–polymerase chain reaction (qRT-PCR) approaches were deployed to assess the involvement of CAMTAs under different abiotic stress conditions. For example, *Zea mays* (corn) *ZmCAMTAs* in drought, cold, salt, and hormonal signaling (Yue et al., 2015); *Fragaria ananassa* (Strawberry) *FaCAMTAs* in heat, cold, salt, and ethylene stress (Leng et al., 2015); *T. aestivum* (wheat) *TaCAMTAs* in cold, salt, heat, and drought stress (Yang et al., 2020); *Citrus sinensis* (sweet orange) *CitCAMTAs* in salinity, dehydration, and hormonal response (Zhang et al., 2019); and *Linum usitatissimum* (Flax) *LuCAMTAs* in drought, low temperature, and light responses (Ali et al., 2020) (discussed in detail in *Recent Discoveries of CAMTAs in Various Crop Plants*).

### CAMTA Transcriptional Response Under Biotic Stress Conditions

Transient Ca^2+^ levels are known to be elevated during pathogen attack (Ma and Berkowitz, 2007), which eventually activates CaM. Similar to the role of CAMTA1 in abiotic stress tolerance, the role of CAMTA3 in response to biotic stress has been elucidated to a great extent (Galon et al., 2008; Laluk et al., 2012; Benn et al., 2016; Kim et al., 2017, 2020; Jacob et al., 2018). Ca^2+^-loaded CaM regulates the activity of CAMTA3 either by negative regulation of CAMTA3 or by positive regulation of a negative repressor TF (Galon et al., 2008). The bacterial pathogen *Pseudomonas syringae* and the fungal pathogen *Botrytis cinerea* did not heavily affect *camta3* in comparison to their effect in control plants. This is suggestive of the fact that CAMTA3 is responsible for suppression of biotic defense responses, which may be primarily achieved either by binding of CAMTA3 with the promoters of suppressed genes or by expressing a TF that is repressed. Transcriptomics approach on *camta3* revealed an attenuated expression of six genes and an enhanced expression of 99 genes out of which 32 genes are related to defense response such as *WRKY33*, *PR1*, *chitinase*, etc. Apart from model plants, the role of CAMTA3 in biotic response was also extended to crop plants. *OsCBT* encoding CaM-binding protein was functionally characterized in plant defense. *Oscbt-1* mutants were significantly tolerant to rice blast fungus *Magnaporthe grisea* and the bacterial pathogen *Xanthomonas oryzae* (Choi et al., 2005; Koo et al., 2009). Furthermore, for the identification of transcriptional network governed by *OsCBT*, transcriptome analysis between wild-type (WT) and *oscbt-1* rice grown in pathogen-free conditions was performed. The analysis revealed 81 up-regulated genes and 200 down-regulated genes. Majority of these genes were implicated in biotic stress responses. The gene expression patterns of fungal pathogen response were also found to be significantly affected in *oscbt-1* mutant, suggesting the probability of *OsCBT* in regulating rice defense response (Chung et al., 2020).

Pathogen attack on plants causes intracellular Ca^2+^ spikes; however, how Ca^2+^ mediates this interaction-based SA level fluctuation remains unclear. CAMTA3 has been reported to link Ca^2+^ transients to SA-mediated immune response. CAMTA3 also interacts with EDS1 promoter to impair its expression (Du et al., 2009). Further research has led to the establishment of the role of CAMTA3 in plant defense and ethylene-induced senescence (Nie et al., 2012). *Edr2* (enhanced disease resistance 2) suppressors were screened to establish the components of EDR2 pathways. This led to the identification of gain-of-function mutation in *signal responsive1* (*SR1*) or CAMTA3. *Edr2* mutants have enhanced tolerance for *Golovinomyces cichoracearum* (powdery mildew), indicating that EDR2 is a negative regulator for powdery mildew. The *sr1-4D* mutation restrains edr2-mediated powdery mildew tolerance. Post mildew infection, as compared to wild type, the *edr2* plants displayed necrotic lesions with little powder. However, the *edr2 sr1-4D* plants phenotypically resemble that of the control plants with oodles of conidia on leaves. The *sr1-4D* gain-of-function also represses elevated ethylene induced senescence. Thus, CAMTA3 negatively impacts plant immune responses. It also directly interacts with the promoter of NDR1 (non-race specific disease resistance1), a key module in resistance to *P. syringae*–mediated plant immunity. Moreover, CAMTA3 also regulates EIN3 (ethylene insensitive3), which contributes to ethylene-induced senescence (Nie et al., 2012).

As discussed previously, CAMTA1, 2, and 3 play pivotal roles in SA-mediated plant immunity. Pip (pipecolic acid) regulates the process of systemic acquired resistance (SAR) in plants (Bernsdorff et al., 2016). CAMTA1, 2, and 3 suppress the synthesis of Pip by inducing ALD1 (AGD2-like defense response protein 1). *ALD1* is known to encode an enzyme required for Pip biosynthesis. The induction of *ALD1* results in an accumulation of Pip, which in turn increases the concentration of NPR1 (SA receptor protein). Thus, CAMTA123 triple mutation induces plant defense machinery and instigates SAR (Kim et al., 2020). Moreover, CBP60g and SARD1 TFs are regulators of SA and NHP (*N*-hydroxypipecolic acid). Both SA and NHP have been extensively linked to plant immunity. CBP60g has been identified as a direct target of CAMTA3 through chromatin immuno-precipitation (ChIP) and electrophoretic mobility shift assay (EMSA) assays. Thus, CAMTAs suppress SA and NHP biosynthesis by regulating SARD1 and CBP60g expression (Lenzoni et al., 2018; Sun et al., 2020).

CAMTA3, as defense against insect herbivory, has also been studied in plants (Qiu et al., 2012). Loss-of-function mutation of *AtSR1* suppresses resistance to feeding by *Trichoplusia ni* (generalist herbivore), sustaining radically elevated larval weight gains and jasmonate (JA) accumulation owing to wound. The responsiveness of *AtSR1* mutant to *T. ni* is attributed to low glucosinolate (GS) levels with remarkably decreased levels in indol-3-ylmethyl (I3M) and 4-methylsulfinylbutyl (4MSOB), the two well-characterized herbivory deterrents. Induction of the various genes implicated in GS metabolism such as *IQD1*, *MYB51*, and *AtST5a* also changes with alterations in *AtSR1* transcript levels. Thus, *AtSR1* through Ca^2+^ signaling cascade acts as a key module of plant resistance to insect herbivory. Additionally, Ca^2+^/CaM-dependent signaling plays a major role in GS metabolism through CAMTA3 involvement (Laluk et al., 2012). Furthermore, upon pathogen exposure, plants allocate their energy resources (at the expense of growth) to defend themselves against invading pathogens. Yuan et al. (2018a) well-documented *AtSR1* as a negative regulator of plant immune responses ( PTI-, ETI-, SA-, and JA-mediated signaling pathways) and a positive regulator of plant growth (auxin and BR signaling pathways). EMSA and ChIP assays demonstrated that *AtSR1* maintains a strong balance between plant immunity and growth by interacting with the CGCG motif present in the upstream sequences of its potential target genes (Yuan et al., 2018a). The same has also been previously established by Li et al. (2014), who identified seven *SISR/CAMTA* genes in tomato (*Solanum lycopersicum*). Functional analysis using VIGS indicated that both *SlSR1* and *SlSR3L* negatively regulate plant defense responses. *SISRs* were explicitly induced upon *P. syringae* pv. tomato (*Pst*) DC3000 and *B. cinerea* infection. The knockouts of *SlSR1* or *SlSR3L* displayed higher tolerance to *Pst DC3000* and *B. cinerea* with subsequent accumulation of H_2_O_2_. Moreover, the genes implicated in pattern-triggered immunity, defense responses, and ethylene and SA pathways were significantly elevated (Li et al., 2014).

**FIGURE 3:**
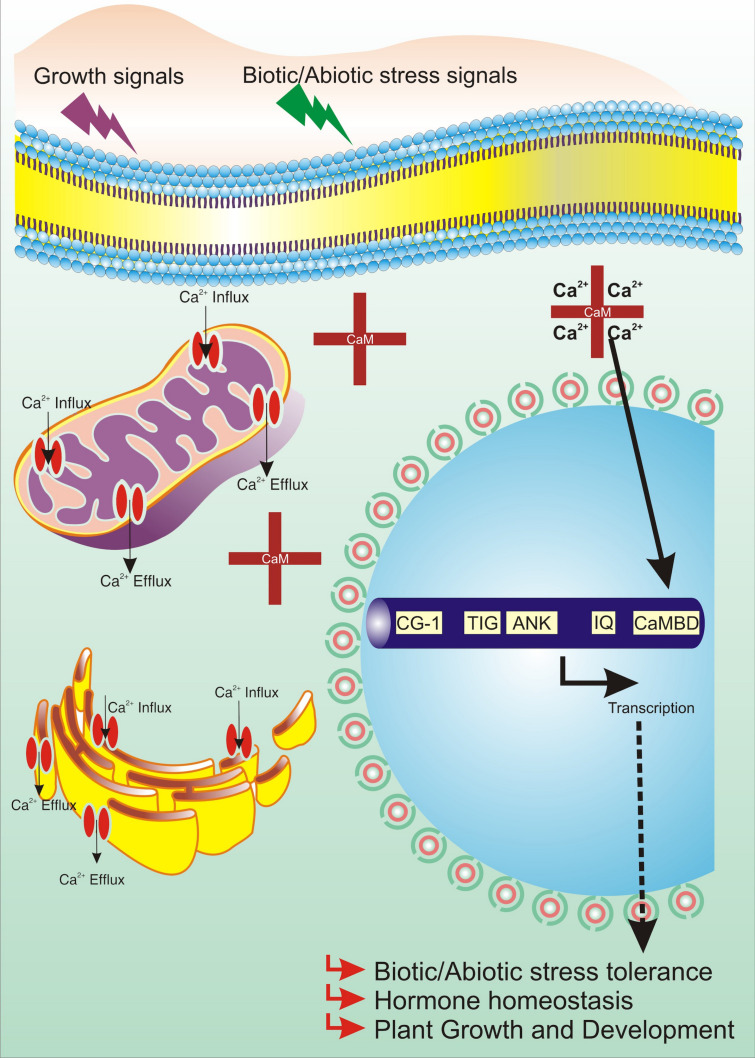
Working model of CAMTA in plant cells.

To cope with environmental constraints, plants have synchronized mechanisms underlying GSR (general stress response) networks and stress-specific networks (Benn et al., 2014). RSRE (rapid stress response element), the functional motif of GSR, has been extensively studied to explore the new horizons of stress signaling (Walley and Dehesh, 2010). CAMTA3 has been very well studied as a pivotal modulator of RSRE-mediated GSR (Bjornson et al., 2014). The function of CAMTA in RSRE response was hypothesized on the basis that Ca^2+^ ion inducers, flagellin22 (flg22) and oligogalacturonic acid, enhance the RSRE activity, and Ca^2+^ ion chelator, EGTA, reduces the RSRE activity upon wounding. CAMTA3 lies downstream of MEKK1, facilitating the regulation of peak time and amplitude of plant GSR and, in conjugation with CAMTA2 and CAMTA4, stimulates RSRE (Benn et al., 2014; Bjornson et al., 2014). Moreover, the methylerythritol cyclodiphosphate (MEcPP)–triggered induction of GSR by transduction of CAMTA3 has also been reported (Benn et al., 2016). CCaMKS and CDPKs all together act to relay the Ca^2+^ signals essential for RSRE activation. Induction of RSRE by Ca^2+^ outburst is in accordance with the defined and established role of this transducer in stress signaling (Dodd et al., 2010). CAMTA3 conceivably has also been hypothesized to negatively regulate PTI (PAMP-triggered immunity) and defense by direct targeting of BAK1 and JIN1 through suppression of JA signaling pathway (Rahman H. et al., 2016). CAMTA3 also controls the transcriptional regulation of early convergence of leucine-rich repeat–containing protein (NLR) and pattern recognition receptor (PRR) signaling. CAMTAs, being a probable target of pathogen effectors, promote pathogen virulence. Furthermore, *camta3* mutants activate NLRs, resulting in host cell death. Deciphering the NLR-driven CAMTA activity underlines the connection of CAMTA in plant innate immunity (Lolle et al., 2017; Jacob et al., 2018). Hence, ever since its discovery, CAMTAs have been implicated in various developmental and cellular processes either themselves acting as a key TF or targeting another TF through direct or indirect association. The overall involvement of CAMTAs in diverse molecular processes is shown diagrammatically in Figure 3.

### Recent Discoveries of CAMTAs in Various Crop Plants

The function of CAMTAs has been established in crop plants like corn (*Z. mays*), strawberry (*F. ananassa*), barrelclover (*Medicago truncatula*), poplar (*Populus trichocarpa*), soybean (*Glycine max*), grapevine (*Vitis vinifera*), tomato (*S. lycopersicum*), and cotton (*Gossypium* spp.), where they were previously and largely unknown (Yang et al., 2013, 2015; Shangguan et al., 2014; Leng et al., 2015; Wang et al., 2015; Yue et al., 2015; Wei et al., 2017; Pant et al., 2018). Nine *ZmCAMTAs* have been reported, upon scanning the entire genome of maize. These CAMTAs were shown to share homology with rice CAMTA. Moreover, an exceedingly high degree of sequence and structural pattern existed among the *ZmCAMTAs*, indicating that their origin is from a single ancestral sequence. All these CAMTAs possess highly tissue-specific expression and display slight diversity in their gene organization. The qRT-PCR of *ZmCAMTAs* under various abiotic stress (drought, salt, and cold), biotic stress (RBSDV-rice black-streaked dwarf virus infection), and stress hormones (ABA, auxin, SA, and MeJA) reveals its role as a stress-responsive gene. The occurrence of many stress-related *cis-*regulatory element in the 1.5-kb promoter region of *ZmCAMTAs* further confirmed its role in stress regulation (Yue et al., 2015). Taking into account tomato (*S. lycopersicum*), a horticulturally important crop, signal-responsive (SR)/CAMTA TF is well implicated in postharvest biology. SlSRs, in response to stress conditions, also coordinate Ca^2+^ signaling with signal transduction pathways implicated in stress during fruit ripening and storage, thus indicating its significant role in horticultural sector (Yang et al., 2013). As previously discussed, SlSRs are involved in plant defense responses and drought management. In *S. lycopersicum*, *SlSR1* and *SlSR3L* negatively regulate plant defenses against *B. cinerea* and *Pst* DC3000. Conversely, *SlSR1L* is involved in the positive regulation of drought stress responses (Li et al., 2014).

In *F. ananassa*, 4 CAMTAs, namely, *FaCAMTA1*, *FaCAMTA3*, *FaCAMTA4*, and *FaCAMTA5*, have been reported, upon the exploitation of BLAST and HMMER tool bioinformatically, using the CAMTA family domain file. All these CAMTAs possessed known domain organization comprising CG1, TIG, ANK repeats, and IQ motifs. Under heat, cold, salt, and ethylene stresses, the *FaCAMTAs* exhibit distinct expression patterns (Leng et al., 2015). *CAMTA* genes have also been identified in *M. truncatula* genome, specifying its role in nodule organogenesis. The *MtCAMTA* genes display tissue-specific expression and respond to various stress-related hormones. Upon *Sinorhizobium meliloti* infection, the expression profiling of the *MtCAMTA* genes revealed that there is an alleviation in the suppression of most *MtCAMTA* genes expression, indicating the CAMTA involvement in early nodulation signaling pathway. Moreover, the promoters of early rhizobial infection response genes display the presence of CAMTA binding motifs (Yang et al., 2015). Similarly, seven CAMTAs were identified in the genome of *P. trichocarpa*, and their expression patterns were analyzed in roots and leaves. The qRT-PCR analysis showed that their expression was induced by pathogenic infection with *Alternaria alternata*; mannitol, NaCl, and cold stress; and phytohormones including SA, ABA, and MeJA (Wei et al., 2017). This was further substantiated as their promoter analysis revealed that most *PtCAMTAs* contain phytohormone or stress-related *cis-*regulatory elements. In *G. max*, 15 *CAMTA* genes were identified, sharing sequence homology with their *Arabidopsis* counterparts. All *GmCAMTAs* were profoundly expressed in root and leaf tissue. Their transcript abundance was induced significantly upon dehydration, cold, salinity, and hormonal treatments such as ABA, SA, and MeJA. In concurrence to their receptiveness to these signals, the promoter regions of these genes have been reported to be enriched with stress-related *cis-*regulatory elements (Wang et al., 2015). Complete functional analysis of *GmCAMTA12*, in relation to drought stress, has been performed. The promoter region of *GmCAMTAs* contained ABRE, SARE, G-box, and W-box *cis-*regulatory elements. *Arabidopsis* T3 overexpression lines of *GmCAMTA12* display enhanced drought tolerance and increased survival and germination rate under drought. *GmCAMTA12* overexpression lines performed better in terms of physiological parameters such as proline and malondialdehyde contents, catalase activity, and electrolyte leakage (Noman et al., 2019). In *V. vinifera*, 10 SR/CAMTAs were identified bioinformatically as Ca^2+^/CaM-binding protein, belonging to four gene groups: *VvCAMTA1*, *VvCAMTA3*, *VvCAMTA4*, and *VvCAMTA5* and localized on 5, 7, 1, and 5 chromosomes, respectively. Expression analysis of *VvCAMTAs* depicted its role mainly in Ca^2+^ signal relay, with significantly high expression in bud, fruit, and inflorescence (Shangguan et al., 2014). Additionally, in four different *Nicotiana* species (*Nicotiana tabacum*, *Nicotiana sylvestris*, *Nicotiana tomentosiformis*, and *Nicotiana benthamiana*), 29 CAMTAs (13 in *N. tabacum*, 6 in *N. sylvestris*, and 5 each in *N. tomentosiformis* and *N. benthamiana*) were identified by evolutionary and expression analyses, which provided great insights into CAMTAs origin, expansion, and response toward stress conditions and plant development (Kakar et al., 2018). *NtabCAMTAs* are among the early responsive genes to biotic stress (*cucumber mosaic virus-*M -M strain of CMV, *potato virus* Y -Mn strain of PVY, and fungal pathogen black shank or *Phytophthora nicotianae*) and played pivotal roles in plant defense. *NtabCAMTAs* were also significantly responsive to abiotic conditions such as cold, drought, and cadmium stress (Kakar et al., 2018).

The identification and functional characterization of CAMTA gene family were also extended to cotton. Six CAMTA members were identified in *Gossypium arboreum*, 7 in *G. raimondii*, and 9 in *G. hirsutum*. Segmental duplication and purifying selection play key roles in the expansion and evolution of CAMTAs in cotton genome. Expression profiling indicated that cotton CAMTAs were predominantly expressed in various stages of fiber development (0, 6, 9 12, and 25 days after anthesis). Precisely, the specific involvement of *GhCAMTA2A.2* and *GhCAMTA7A* in maintaining fiber strength has been reported (Pant et al., 2018). CAMTA gene family has also been classified in *Phaseolus vulgaris* (common bean). *P. vulgaris* contained 11 chromosomes, of which five chromosomes harbored eight *CAMTA* genes with 11 to 12 introns per gene. *Pvul*CAMTAs shared similarity with *Gm*CAMTAs owing to similar genome organization. *In silico* analysis revealed that *Pvul*CAMTAs are involved in salt stress signaling and were further substantiated through RNA-seq and qRT-PCR analyses (Buyuk et al., 2019). In concurrence to the above study, *Pvul*CAMTA1 has also been characterized to impart drought tolerance (Saeidi et al., 2019). *CAMTA* gene family had also been well reported in citrus (Zhang et al., 2019). Nine *CAMTA* genes were found in the citrus (*C. sinensis* and *Citrus clementina*) genomes. Most of the *CitCAMTAs* were found to be highly conserved during evolution. All *CitCAMTA* genes (except *CitCAMTA4*) were expressed in minimum one plant tissue corroborating their involvement in leaf, root, stem, seed, cotyledon, and fruit development. Hormone and stress experiments revealed varying expression profiles of *CitCAMTAs*, indicating stress adaptation. Upon salt stress (0- to 24-h treatment), *CitCAMTA1, CitCAMTA5*, and *CitCAMTA9* were significantly expressed. The expression of *CitCAMTA7* first increased and then decreased as the time of salt treatment progressed. The expression of *CitCAMTA3* was suppressed at all time points. For the remaining *CitCAMTAs*, i.e., *CitCAMTA2*, *CitCAMTA6*, and *CitCAMTA8*, there were no obvious changes observed. Upon dehydration stress (0- to 12-h treatment), *CitCAMTA5*, *CitCAMTA6*, and *CitCAMTA8* levels slightly declined, whereas for *CitCAMTA3* and *CitCAMTA9*, the expression levels were up-regulated reaching a maximum at 12 h of treatment. *CitCAMTA1* and *CitCAMTA2* showed moderate level of expression under dehydration stress. While most of the *CitCAMTAs* were responsive to salt and dehydration stress, *CitCAMTA4* remain unaltered. Lastly, the authors investigated the involvement of *CitCAMTAs* in hormonal signaling (SA, ETH, IAA, 6- BA, ABA, MeJA, and GA3). All the *CitCAMTAs* (except *CitCAMTA4*) were responsive to at least one phytohormone. *CitCAMTA1*, *CitCAMTA5*, *CitCAMTA7*, and *CitCAMTA9* positively correlated to all the hormones, whereas *CitCAMTA3* showed a negative correlation. These observations led the authors to hypothesize the involvement of *CitCAMTAs* in abiotic stress pathways and hormonal regulation (Zhang et al., 2019). Genome-wide identification also led to the identification of 15 wheat *CAMTA* genes, which were classified into three groups A, B, and C containing seven, six, and two *TaCAMTAs*, respectively (Yang et al., 2020). Most of the *TaCAMTAs* contained stress-responsive *cis-*regulatory elements. All the 15 *TaCAMTAs* expressed differentially in various tissues and under different abiotic stresses. The expression analysis of *TaCAMTA1-A* and *1-D* revealed that they were significantly expressed under drought, cold, heat, and salt stress. *TaCAMTA* genes belonging to the same homologous group had similar expression profiles under particular stress condition. For example, *TaCAMTA1-A/B/D* had almost similar expression profile under drought stress, *TaCAMTA5-A/D* under salt stress, and *TaCAMTA1-A/B/D* under heat stress. This observation implies that homologous *TaCAMTA* genes from the same group might share same functionality. However, few homologous *TaCAMTA* genes belonging to the same group displayed varying expression profiles under drought, cold, heat, and salinity stress. For instance, *TaCAMTA3-A/D* was significantly expressed upon cold stress, but the expression profiles of *TaCAMTA1-B* and *TaCAMTA3-B* were comparatively stable. This might be due to the functional differentiation in some homologous *TaCAMTA* genes. Additionally, in the study, it was depicted that 584 genes in the wheat genome can be potential targets of *TaCAMTA*, indicating the probable role of CAMTA in stress biology and plant development (Yang et al., 2020). Furthermore, *TaCAMTA4* had been demonstrated to function as negative regulator of defense response against *Puccinia triticina* (Wang et al., 2019). *TaCAMTA4* was found to be homologous to *AtCAMTA4* and has been very well implicated as a CaM-binding protein in the wheat–*P. triticina* interaction system. This was proved by cloning and functionally characterizing *TaCAMTA4* by using the CaM-encoding gene (*TaCaM4-1)* as a bait, for subsequent screening of cDNA library from *P. triticina*–infected *Triticum* leaves. The EMSA results revealed that TaCaM4-1 binds to TaCAMTA4 by the C-terminal CaMBD in Ca^2+^-dependent manner. Bimolecular fluorescence complementation (BiFC) analysis showed cytoplasm and nucleus as the probable interaction site of TaCAMTA4 and TaCaM4-1 (Wang et al., 2019). In a similar vein, nine CAMTA genes were identified in Flax (*L. usitatissimum*) upon genome-wide identification study. These nine CAMTA proteins were classified into three groups, depending on the phylogenetic analysis. Various hormonal (ABA and SA) and stress-related (drought, low temperature, and light) *cis-*regulatory elements were found to be enriched in promoter region of *LuCAMTAs* genes. In addition, the miRNA target analysis showed different miRNA families (miRN30, miRN9, miR2275, miR164, miR159, miR164, miRN15, miR395, miR156, miRN28, and miR164) as probable targets of the *LuCAMTAs* genes (except *LuCAMTA9*) (Ali et al., 2020).

## Conclusion and Future Perspective

Improving crop yield and productivity for the ever-growing population has been a major challenge all over the world. The demand for ever-increasing food supply has put forth a tremendous pressure on the agriculture sector. The advent of new biotechnological tools and techniques has robustly contributed to crop improvement and horticultural science (Limera et al., 2017; Adlak et al., 2019). In this regard, several TFs, including CAMTA, had been identified and functionally characterized. Ever since the discovery of CAMTAs, its role had been comprehensively reported in developmental and stress biology in various model and crop plants (Table 1). The objective ahead is to understand the fundamental functionality and molecular mechanism behind CAMTA protein expression and downstream processing. A deeper understanding of the mechanisms underlying the involvement of Ca^2+^ and CaM in *CAMTA* transcript expression would provide great insights into its probable function in various tissues and response to environmental cues. In addition, deciphering the probable functions and mechanisms underlying the complex regulatory property of CAMTA proteins through its ability to transduce Ca^2+^ signatures via CaM can improve our knowledge of phenotypic plasticity in plants. However, because of the possible functional redundancy or at least overlapping functions, molecular characterization of individual *CAMTA* genes still poses a great challenge ahead for researchers. Rigorously developed CRISPR/Cas9-mediated genome editing techniques that rapidly generate mutations at multiple loci using one single-guide RNA will effectively help characterizing each of the gene family members. Nonetheless, the involvement of *CAMTA* as stress-related gene and its subsequent study in this direction would widen and provide vast knowledge for improvement in crop yield, productivity, and strategies to cope with adverse conditions in arid or semiarid regions of the world.

**TABLE 1:** Reported functions of CAMTA in plants.

Gene name	Function	Source organism	Reference
*AtCAMTA1*	Drought tolerance via ABA signaling	*Arabidopsis thaliana*	Pandey et al., 2013
	Cold acclimatization	*A. thaliana*	Doherty et al., 2009
	Repressor of ICS1, CBP60g, and SARD1 (suppressor of SA accumulation)	*A. thaliana*	Kim et al., 2013; Sun et al., 2020
	Salt stress response	*A. thaliana*	Galon et al., 2010a
	Auxin homeostasis, transport, and signaling	*A. thaliana*	Galon et al., 2010a, b
	Pipecolic acid biosynthesis and priming of immunity genes	*A. thaliana*	Kim et al., 2020
*AtCAMTA2*	Suppressor of SA biosynthesis-related gene transcripts	*A. thaliana*	Kim et al., 2013; Sun et al., 2020
	Activator of *AtALMT1* (metal toxicity)	*A. thaliana*	Tokizawa et al., 2015; Wu et al., 2019
	Pipecolic acid biosynthesis and priming of immunity genes	*A. thaliana*	Kim et al., 2020
*AtCAMTA3*	Activator of RSRE	*A. thaliana/Nicotiana benthamiana*	Benn et al., 2014
	Positive regulator of CBF2	*A. thaliana*	Doherty et al., 2009
	Suppressor of SA biosynthesis	*A. thaliana*	Kim et al., 2013; Sun et al., 2020
	Suppressor of plant defense responses	*A. thaliana*	Galon et al., 2008; Du et al., 2009
	Plant defenses against insect herbivory via SA-JA crosstalk	*A. thaliana*	Laluk et al., 2012; Qiu et al., 2012
	Ethylene-induced senescence	*A. thaliana*	Nie et al., 2012
	Negative regulation of resistance to *S. sclerotiorum*	*A. thaliana*	Rahman H. et al., 2016
	NLR- and PRR-mediated signaling	*A. thaliana*	Jacob et al., 2018
	MEcPP mediated induction of the rapidly and transiently stress-responsive *cis-*element	*A. thaliana*	Benn et al., 2016
	Glucosinolate metabolism and herbivory tolerance	*A. thaliana*	Laluk et al., 2012
	Freezing tolerance	*A. thaliana*	Kim et al., 2013
	Salicylic acid immunity to low temperature and pathogen infection	*A. thaliana*	Kim et al., 2017
	Regulation of peak time and amplitude of the plant GSR	*A. thaliana*	Bjornson et al., 2014
	Plant death and senescence	*Nicotiana tabacum*	Yang and Poovaiah, 2000
	Pipecolic acid biosynthesis and priming of immunity genes Regulate *DREB1B* upon rapid decrease in temperature Negative regulator of plant immune responses and positive regulator of plant growth	*A. thaliana A. thaliana A. thaliana*	Kim et al., 2020 Kidokoro et al., 2017 Yuan et al., 2018a
*AtCAMTA4*	Positive regulator of auxin homeostasis	*A. thaliana*	Galon et al., 2010b
*AtCAMTA5*	BZR1-associated protein; BR signaling	*N. benthamiana*	Wang et al., 2013
	V-PPase expression in pollen Regulate *DREB1B* upon rapid decrease in temperature	*A. thaliana A. thaliana*	Mitsuda et al., 2003 Kidokoro et al., 2017
*AtCAMTA6*	Na^+^ homeostasis in seed germination	*A. thaliana*	Shkolnik et al., 2019
*OsCBT*	The negative regulator on plant defense Fungal pathogen response	*Oryza sativa O. sativa*	Choi et al., 2005; Koo et al., 2009 Chung et al., 2020
*SlSR1, SlSR3L*	Negative regulators of defense response against *B. cinerea* and Pst DC3000	*Solanum lycopersicum*	Li et al., 2014
*SlSR1L*	Positive regulator of drought stress	*S. lycopersicum*	Li et al., 2014
*SlSRs*	Regulation of SA levels during fruit ripening and development	*S. lycopersicum*	Yang et al., 2013
*VvCAMTA1; VvCAMTA3; VvCAMTA4; VvCAMTA5*	Ca^2+^ signal transduction	*Vitis vinifera*	Shangguan et al., 2014
*GmCAMTA;*	Responsive to stress and hormone signals	*Glycine max*	Wang et al., 2015
*GmCAMTA12*	Drought tolerance	*G. max*, *A. thaliana*	Noman et al., 2019
*MtCAMTAs*	Nodule organogenesis	*Medicago truncatula*	Yang et al., 2015
*ZmCAMTA*	Biotic/abiotic stress tolerance	*Zea mays*	Yue et al., 2015
*PtCAMTA*	Biotic/abiotic stress management and ABA, SA, MeJA homeostasis	*Populus trichocarpa*	Wei et al., 2017
*GhCAMTA2A.2; GhCAMTA7A*	Cotton fiber development	*Gossypium hirsutum*	Pant et al., 2018
*FaCAMTA*	Heat, cold, and salt stress	*Fragaria ananassa*	Leng et al., 2015
*BnCAMTA*	Stress-inducible and phytohormonal regulation	*Brassica napus*	Rahman H. et al., 2016
*NtabCAMTAs*	Drought, cold, cadmium, CMV, PVY, and black shank stress	*N. tabacum*	Kakar et al., 2018
*PvulCAMTA*	Drought	*Phaseolus vulgaris*	Buyuk et al., 2019; Saeidi et al., 2019
*TaCAMTAs;*	Drought, cold, heat, and salinity	*Triticum aestivum*	Yang et al., 2020
*TaCAMTA4*	Negative regulator of defense response against *P*. *triticina*	*T. aestivum*	Wang et al., 2019
*CitCAMTAs*	Salt, dehydration, and hormone stress	*Citrus sinensis* and *Citrus clementina*	Zhang et al., 2019
*LuCAMTAs*	ABA, SA, drought, low temperature and light responsive	*Linum usitatissimum*	Ali et al., 2020

## Author Contributions

ZI drafted the manuscript. MSI helped in drawing the figures. SPS edited the manuscript. TB reviewed the manuscript. All authors contributed to the article and approved the submitted version.

## Conflict of Interest

The authors declare that the research was conducted in the absence of any commercial or financial relationships that could be construed as a potential conflict of interest.
